# Chemoresistance is associated with Beclin-1 and PTEN expression in epithelial ovarian cancers

**DOI:** 10.3892/ol.2015.2950

**Published:** 2015-02-10

**Authors:** HUANCHUN YING, DONGHUI QU, CHUAN LIU, TIANSHU YING, JING LV, SHANSHAN JIN, HONGYING XU

**Affiliations:** 1 Department of Gynecology and Obstetrics, Shengjing Hospital of China Medical University, Shenyang, Liaoning 110004, P.R. China; 2 Department of Gynecology and Obstetrics, Affiliated Hospital of Chengde Medical College, Chengde, Hebei 067000, P.R. China; 3 Department of Oncology, The Fifth Hospital of Shenyang, Shenyang, Liaoning 110023, P.R. China; 4 Department of Gynecology and Obstetrics, The Ninth Hospital of Shenyang, Shenyang, Liaoning 10024, P.R. China

**Keywords:** Beclin-1, phosphatase and tensin homolog, ovarian cancer, platinum, resistant, autophagy

## Abstract

The aim of the present study was to investigate the protein expression of the autophagy-related genes, *BECN1* and *PTEN*, and the association with drug resistance in epithelial ovarian cancers. In total, 40 patients with pathologically diagnosed epithelial ovarian cancer were divided into a chemotherapy-sensitive group (n=20) and a chemotherapy-resistant group (n=20), according to the results of the pre- or post-operative normative chemotherapy and the post-operative follow-up. The protein expression of the phosphatase and tensin homolog (PTEN) and the *BECN1* gene product, Beclin-1, was analyzed using immunohistochemistry in the 40 patients with ovarian carcinoma. The positive rate of Beclin-1 expression was significantly lower in the resistant group (35.0%) compared with the sensitive group (50.0%). The positive rate of PTEN expression was also significantly lower in the resistant group (30.0%) compared with the sensitive group (65.0%). Furthermore, the differences in the expression rates were revealed to be significant (P<0.05). The expression of Beclin-1 was identified to be positively correlated with the expression of PTEN (rs=0.816; P<0.0001). The low expression of the Beclin-1 and PTEN proteins in the ovarian cancer tissues was revealed to be closely associated with drug resistance. Therefore, Beclin-1 may interact with PTEN to participate in the mechanism of drug resistance and the changes in macrophage activity observed in cases of drug-resistant ovarian cancer.

## Introduction

Ovarian cancer is a gynecological malignancy with a high rate of mortality. Due to a lack of notable symptoms in the early stages, 60% of patients present with advanced-stage disease at diagnosis (1). The primary clinical treatment for ovarian cancer is cytoreductive surgery combined with platinum-based chemotherapy. Chemotherapy is a significant part of the radical treatment approach for ovarian cancers, however, platinum-based chemotherapies are prone to drug resistance; a factor that affects treatment efficacy and contributes to poor prognoses. At present, the five-year survival rate for ovarian cancer is 30–50% (2). Therefore, in order to improve the prognoses of these patients, the issue of drug-resistance must be addressed. Previous studies have identified that the abnormal regulation of autophagy is directly associated with tumorigenesis (3–5). Therefore, autophagy has become a novel target for the investigation of tumorigenesis, the inhibition of tumor growth and for determining how to overcome chemotherapy-associated drug resistance. However, the mechanism involved in the regulation of autophagy is complex.

*BECN1*, an autophagy-related gene located on human chromosome 17q21, encodes the protein Beclin-1, which participates in the formation of phages and can inhibit tumor growth by increasing the rate of autophagy. *BECN1* has therefore been identified as a candidate tumor suppressor gene for ovarian cancer (6). Previous studies have revealed that the expression of Beclin-1 is downregulated in breast (7), prostate (8) and nasopharyngeal (9) carcinomas. Further studies have revealed that a loss of Beclin-1 expression results in reduced autophagy activity and increased tumorigenesis (10). The *PTEN* gene, located on chromosome 10, is a tumor suppressor gene with dual specificity phosphatase activity. The phosphatase and tensin homolog (PTEN) protein is involved in the regulation of a variety of cellular signal transduction pathways, and is closely associated with cell growth and differentiation, and with tumorigenesis. Furthermore, PTEN is able to initiate autophagy and therefore inhibit tumor growth. In the present study, the protein expression of Beclin-1 and PTEN in ovarian cancer tissues was detected using immunohistochemistry. The study aimed to investigate the role of Beclin-1 and PTEN, and the association with resistance in ovarian cancer. The effect of autophagic changes in cases of drug-resistant ovarian cancer was also examined, in order to further elucidate on the mechanism of drug resistance and aid in the eventual overcoming of ovarian cancer.

## Materials and methods

### Patient characteristics

In total, 40 tissue samples were obtained from patients with ovarian cancer who had undergone cytoreductive surgical resection at the Shengjing Hospital of China Medical University (Shenyang, China) between January 2007 and May 2012. The cases were classified as serous carcinoma (n=24), mucinous carcinoma (n=8), clear cell carcinoma (n=4), endometrial carcinoma (n=2) or undifferentiated carcinoma (n=2). The age range of the patients was 35 to 58 years old, with a median age of 48 years old. The pathological surgical staging was performed according to the 2009 International Federation of Gynecology and Obstetrics system as follows: Stage I, two cases; stage II, 10 cases; stage III, 24 cases; and stage IV, four cases (11).

The telephone or hospital examination follow-ups of the 40 cases of ovarian cancer began at diagnosis and ended on December 31, 2012. The patients were pathologically diagnosed with ovarian cancer and divided into a chemotherapy-sensitive (n=20) or chemotherapy-resistant (n=20) group, according to the results of the pre- or post-operative normative chemotherapy and the post-operative follow-up. The grouping standards were as follows: i) Achievement of clinical remission following the initial platinum-based chemotherapy was necessary for study inclusion; and ii) cases with relapse at six months or more following the end of the chemotherapy program were taken as the chemotherapy-sensitive group, and cases with relapse within six months were used as the chemotherapy-resistant group. This study was approved by the ethics committee of Shengjing Hospital of China Medical University and written informed consent was obtained from all patients.

### Immunohistochemical analysis

The Beclin-1 polyclonal rabbit (1:100 dilution) and PTEN monoclonal mouse (1:75 dilution) antigen solutions were purchased from Proteintech (Chicago, IL, USA). The immunohistochemistry Power Vision kit and 3,3′-diaminobenzidine reagent were purchased from Beijing Golden Bridge Zhongshan Biotechnology Co., Ltd. (Beijing, China).

Phosphate-buffered saline solution was used for the negative control, and normal known-positive ovarian tissue samples were used for the positive control. Using the Power Vision kit, the experimental procedure was performed according to the manufacturer’s instructions. A total of 10 randomly selected high-magnification fields were analyzed under an optical microscope (AX70; Olympus Corporation, Tokyo, Japan) 48 h after sealing. PTEN-positive cells were identified by clear-brown granules located in the nucleus or cytoplasm, and Beclin-1-positive cells were identified by brown particles distributed throughout the cytoplasm. The percentage of positive cells to total cells was counted and awarded points according to the following criteria: <10%, 0 points; 10–20%, 1 point; 21–50%, 2 points; and >50%, 3 points. The intensity of staining was also awarded points according to the following system: no color, 0 points; pale yellow, 1 point; brown, 2 points; and tan, 3 points. The total score for each case was the sum of the points for the percentage of positive cells and the staining intensity. A score of ≤3 was regarded as negative for PTEN/Beclin-1 expression, whereas a score of >3 was regarded as positive for PTEN/Beclin-1 expression.

### Statistical analysis

The statistical analysis was performed using SPSS 11.0 (SPSS, Inc., Chicago, IL, USA). The association between the clinicopathological parameters and the protein expression of Beclin-1 and PTEN was analyzed using Student’s t-test. The Spearman’s ρ test was used for the correlation analysis.

## Results

**Figure 1:**
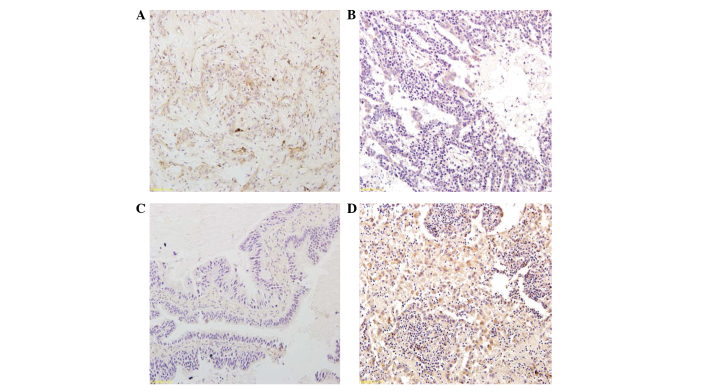
Immunohistochemistry revealing clear-brown nuclear and cytoplasmic granules in phosphatase and tensin homolog (PTEN)-positive cells, and brown cytoplasmic particles in Beclin-1-positive cells (magnification, ×200). Chemotherapy-sensitive group; (A) PTEN and (B) Beclin-1. Chemotherapy-resistant group; (C) PTEN and (D) Beclin-1.

In the 40 cases of ovarian cancer included in the present study, the expression of Beclin-1 was revealed to be primarily located in the cytoplasm. The positive rate of Beclin-1 expression was significantly lower in the chemotherapy-resistant group (35.0%) compared with the chemotherapy-sensitive group (50.0%) (P<0.05). The expression of PTEN was identified to be primarily located in the nucleus or cytoplasm. The positive rate of PTEN expression was significantly lower in the chemotherapy-resistant group (30.0%) compared with the chemotherapy-sensitive group (65.0%) (P<0.05; Table I; Fig. 1).

The correlation analysis revealed that the intensity of Beclin-1 expression was positively correlated with the expression of PTEN in the 40 cases of ovarian cancer (P<0.05; Table II).

## Discussion

Autophagy is a lysosomal degradation process for cellular macromolecules and damaged organelles, and an alternative form of programmed cell death to apoptosis in eukaryotes. Changes in the activity of signaling, transport and negative regulatory pathways of macrophages have been revealed to be associated with tumor occurrence and development. Furthermore, the abnormal expression of autophagy genes has been identified to activate or inhibit the formation of certain tumors (12).

The PTEN protein is encoded by the *PTEN* tumor suppressor gene located on chromosome 10 (13). Since the discovery of the gene in 1997, *PTEN* has gained particular attention for its role in cancer (14). Previous studies have demonstrated that the gene is associated with endometrial (15) and prostate cancers (16), and with malignant gliomas (17). According to the literature, genetic alterations in *PTEN*, such as mutation, loss of heterozygosity, hypermethylation, microsatellite instability or translational modifications result in the ‘silencing’ of gene expression (18). Previous studies revealed that the inhibition of autophagy was removed by inhibiting the change of PTEN 4,5-phosphatidylinositol diphosphate (PIP2) to 3,4,5-triphosphate phosphatidylinositol (PIP3) to participate in autophagy regulation. It has been demonstrated that *PTEN* and other autophagy-related genes are expressed in normal ovarian tissues, benign ovarian tumors and borderline ovarian tumors, but are downregulated in cases of ovarian cancer. This suggests that the decreased expression of autophagy-related proteins, such as PTEN, may be closely correlated with the development of ovarian cancer (19,20). In addition, PTEN protein expression has been identified to be positively correlated with the differentiation state of ovarian cancers (21). A previous study demonstrated that a chemical, EF24, conferred sensitivity to drug-resistant ovarian cancer cells via the upregulation of PTEN expression (22). Furthermore, Wu *et al* (23) upregulated PTEN protein expression in a cisplatin-resistant ovarian cancer C13K cell line via the *in vitro* liposomal transfection of the *PTEN* gene. The C13K cells exhibited increased sensitivity to cisplatin-induced apoptosis compared with empty vector-transfected cells. An *in vitro* study by Yan *et al* (24) demonstrated that PTEN protein overexpression increased the sensitivity of the chemoresistant cell lines, CI3* and A2780cp, to cisplatin-induced apoptosis through the upregulation of p53, rather than the inhibition of Akt protein activation. It was further revealed that low PTEN expression was apparent in the OVCAR-3/CDDP drug-resistant ovarian cancer cell line compared with the normal OVCAR-3 ovarian cancer cell line. The phosphoinositide 3-kinase (PI3K)/Akt pathway is an important pathway involved in the regulation of autophagy, and has been revealed to be inhibited by PTEN. The study identified that PTEN protein expression was significantly lower in the drug-resistant ovarian cancer tissues compared with the drug-sensitive group. This suggested that PTEN was involved in the mechanism of drug resistance in ovarian cancer tissues via the process of autophagy.

The ~150-kb human *BECN1* gene is a homolog of the atg6/vps30 yeast gene and is located on chromosome 17q21. *BECN1* has been revealed to be involved in the development of tumors by the regulation of autophagy (25). At present, autophagy as a barrier for impeding tumorigenesis or tumor adaptive responses remains controversial (26). On the one hand, tumor cells are often restricted due to rapid growth, and therefore autophagy offers an adaptive response for tumor cell survival. On the other hand, certain studies have demonstrated that tumor progression is inhibited by the stimulation of non-apoptotic cell death processes, namely autophagy (27). Therefore, autophagy both inhibits tumor formation and promotes tumor development, and this association remains controversial. Recently, the dynamic role of autophagy in cancer was proposed by Kimmelman (28). The study suggested that autophagy initially acts as a barrier to prevent the initiation of tumors, but that following the formation of lesions, it positively affects malignancy and tumor maintenance. A recent study revealed that Beclin-1 expression levels differed between types of tumor cell. The expression of Beclin-1 was identified to be downregulated in a variety of tumor cells, such as those of breast, ovarian and prostate cancers, and gliomas (4). Despite this, high levels of autophagy activity were maintained in other cancer cells. In a study by Ahn *et al* (29), samples from 103 patients with colorectal cancer, and 60 with gastric cancer, were immunohistochemically analyzed. Beclin-1 expression was observed in 95 and 83% of the colorectal and gastric cancer samples, respectively. By contrast, little or no expression was revealed in the normal gastric and colorectal tissues. Tang *et al* (30) identified that the mRNA and protein expression levels of Beclin-1 were upregulated in hepatitis B virus-mediated liver cancer, which suggested a role for Beclin-1 in tumor formation. The present study revealed that Beclin-1 protein expression was significantly lower in the drug-resistant group of ovarian cancer tissues compared with the drug-sensitive group. Furthermore, the differences in expression were significant, which suggested that decreased Beclin-1 expression, and the activity of macrophages, may be associated with chemotherapy resistance and poor prognoses in patients with ovarian cancer. Conversely, an upregulation in the expression of the Beclin-1 protein may increase the sensitivity of ovarian cancers to chemotherapy, which could improve treatment efficiency and prognoses.

The present study indicated that Beclin-1 and PTEN may be co-involved in the regulation of autophagy, and therefore affect the occurrence and development of cancer. Autophagic protein turnover is regulated by type I and III PI3Ks. Type I PI3K, and the downstream signal conversion components Akt and target of rapomycin, can inhibit autophagy. PTEN induces autophagy by negative regulation of the type I PI3K. By contrast, the type III PI3K is necessary for the autophagic formation of lysosomal vacuoles. Beclin-1 regulates autophagic activity by modulating the precursor structure of Apg proteins, primarily by the formation of complexes with the type III PI3K. Therefore, Beclin-1 and PTEN may possess similar roles in the self-regulation of macrophage activity (31). The results of the present study revealed that the protein expression of Beclin-1 and PTEN exhibited a significant correlation. This indicated that the proteins may participate together in the mechanism of drug resistance observed in platinum-resistant ovarian cancers.

In summary, the protein expression of Beclin-1 and PTEN was downregulated, which suggested that a decrease in autophagic activity may be associated with drug-resistant ovarian cancers. At present, the specific mechanisms that regulate autophagy remain unclear. Further study will aid in clarifying the role of autophagy, which may provide novel solutions for treating chemotherapy-resistant ovarian cancers, and establish a reliable theoretical basis for the development of novel drugs.

The expression of the Beclin-1 and PTEN proteins, in drug-resistant and drug-sensitive ovarian cancers, was detected using immunohistochemistry and analyzed for potential correlations. The results revealed that Beclin-1 and PTEN protein expression was significantly lower in the chemotherapy-resistant group compared with the chemotherapy-sensitive group. Furthermore, the difference in expression was identified to be significant and positively correlated. The results suggested that Beclin-1 and PTEN protein expression decreased in the drug-resistant ovarian cancer tissues. Therefore, it was concluded that the occurrence of drug resistance in ovarian cancers was closely associated with a low expression of PTEN and Beclin-1. In conclusion, a reduction in autophagic activity, induced by the interaction between Beclin-1 and PTEN, may lead to drug resistance in cases of ovarian cancer.

## Figures and Tables

**Table I:** Expression of Beclin-1 and PTEN in chemoresistant and chemosensitive groups.

		Beclin-1			PTEN		
			
Group	n	−	+	% positive	−	+	% positive
Resistant	20	13	7	35.0	14	6	30.0
Sensitive	20	10	10	50.0	7	13	65.0

PTEN, phosphatase and tensin homolog.

**Table II:** Association between Beclin-1 and PTEN expression.

	Beclin-1	
	
Protein	+	−
PTEN		
+	12	5
−	7	16

PTEN, phosphatase and tensin homolog.
